# Cultural Orientation of Self-Bias in Perceptual Matching

**DOI:** 10.3389/fpsyg.2019.01469

**Published:** 2019-06-28

**Authors:** Mengyin Jiang, Shirley K. M. Wong, Harry K. S. Chung, Yang Sun, Janet H. Hsiao, Jie Sui, Glyn W. Humphreys

**Affiliations:** ^1^Department of Psychology, University of Oxford, Oxford, United Kingdom; ^2^Department of Psychology, University of Hong Kong, Pokfulam, Hong Kong; ^3^Tsinghua University, Beijing, China; ^4^Department of Psychology, University of Bath, Bath, United Kingdom

**Keywords:** cross-culture comparison, independent and interdependent, self-construal, perceptual matching, self-bias

## Abstract

Previous research on cross-culture comparisons found that Western cultures tend to value independence and the self is construed as an autonomous individual, while Eastern cultures value interdependence and self-identity is perceived as embedded among friends and family members (Markus and Kitayama, 1991). The present experiment explored these cultural differences in the context of a paradigm developed by Sui et al. (2012), which found a bias toward the processing of self-relevant information using perceptual matching tasks. In this task, each neutral shape (i.e., triangle, circle, square) is associated with a person (i.e., self, friend, stranger), and faster and more accurate responses were found to formerly neutral stimuli tagged to the self compared to stimuli tagged to non-self. With this paradigm, the current study examined cross-cultural differences in the self-bias effect between participants from Hong Kong and the United Kingdom. Results demonstrated a reliable self-bias effect across groups consistent with previous studies. Importantly, a variation was identified in a larger self-bias toward stranger-associated stimuli in the United Kingdom participants than the Hong Kong participants. This suggested the cultural modulation of the self-bias effect in perceptual matching.

## Introduction

The “self” is an important concept that has been the focus of different fields, from social psychology, cross-culture psychology to social cognitive neuroscience. Many have attempted to decode and explain what the self is in the mind and brain. In recent years, much literature has focused on cultural differences between the East (e.g., East Asia) and the West (e.g., North America and Western Europe). These differences have been referred to as individualism versus collectivism by Triandis (1989), or independence versus interdependence by Markus and Kitayama (1991), and the concept of the self in relation to others is one of the key distinctions between the East and the West (Gardner et al., 1999). This study investigates the impact of cultural experiences on the self-bias effect in British and Hong Kong participants.

**FIGURE 1:**
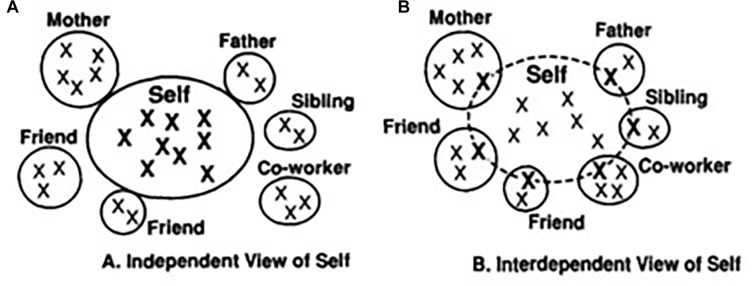
Self-identity in relation to others, as described by Markus and Kitayama (1991). **(A)** Independent self. **(B)** Interdependent self. Xs indicates one’s representations of the self.

Cultural experiences play a crucial part in forming the concept of the self. The self is developed through interactions with, not only other people, but also the social environment and cultural background (Markus and Kitayama, 2003; Kitayama et al., 2007). Markus and Kitayama (1991) stress the importance of independence and interdependence in self-identity. In particular, Western cultures tend to emphasize independence and perceive the self as distinct autonomous entities unique from other people. This encourages one to discover and express one’s unique attributes. In order to achieve independence from other people, one’s self-identity is formed with reference to one’s own internal thoughts, feelings, motivations, and actions irrespective of other people (see Figure 1A). Eastern cultures, however, place greater emphasis on interdependence, which refers to the interconnectedness of oneself to other people and group membership forms an important part of self-identity. This is reflected in the overlap of one’s concept of the self to other people (see Figure 1B).

Typically, one’s level of independence and interdependence is measured through self-reported questionnaires such as the Self-Construal Scale (Singelis, 1994) and the Individualism and Collectivism Scale (Singelis et al., 1995). The focus of these questionnaires is to measure one’s relationship with others and the extent to which representations of the self are perceived as separated or connected with family and friends (Markus and Kitayama, 1991).

Cultural differences not only influence the concept of the self but also affect one’s perception and cognition. East Asians tend to live in a highly interdependent society where attention is directed more to relationships with other people. Westerners, however, live in a more independent society where less attention is paid to the social context. This cultural difference impacts the way stimuli are perceived in the environment. Many have found that Westerners pay more attention to salient objects than contextual background and that Easterners detect contextual relations more than specific items (Nisbett and Miyamoto, 2005). For example, Masuda and Nisbett (2001) demonstrated this attention difference by asking the participants to view and describe a series of vignettes and pictures. In one study, American students tend to describe the most salient objects first (e.g., “I saw a trout”) while Japanese students described the context first (e.g., “I saw a stream”). Results demonstrated that the Japanese students noticed 60% more details about the context than the American students (Masuda and Nisbett, 2001). In another study, American and East Asian participants viewed pictures and vignettes that have focal object or contextual information changes. Findings showed that American participants were more sensitive to changes in focal objects than to changes in the periphery or context. In contrast, East Asian participants were more sensitive to contextual changes than to focal object changes (Masuda and Nisbett, 2006). Similarly, Singelis and Brown (1995) reported that the context of the situation easily influences the behavior of interdependent individuals but not independent individuals. Kühnen et al. (2001) also found that stimulus processing is more affected by context in interdependent than independent self-construals. These findings confirm that culture guides the perception of the self, as well as the way we perceive our environment.

Similar results were also reported by Kitayama et al. (2003) through a framed-line test. Participants viewed a square with a line inside and were asked to reproduce the line in another square in either the same absolute length or in the same proportion to the square. The East Asian participants performed more accurately in the relative task than the absolute task. In contrast, the Western participants performed better in the absolute task than the relative task. The Westerners were also much better at the absolute task than the East Asians, while the East Asians outperformed the Westerners in the relative task.

Eye movement study also demonstrated culturally different viewing patterns. Objects placed into photographs of naturalistic scenes were presented to the participants for viewing. It was found that Americans fixated more on the object, whereas the Chinese spent more time looking at the background (Chua et al., 2005). Thus, the context of the situation heavily influences stimuli processes and the responses of the interdependent individuals (Singelis and Brown, 1995; Kühnen et al., 2001).

As a result of the focus on contextual information, people from interdependent cultures have adapted to perceive the self and close others very differently from independent cultures. Memory encoded in self-reference had been found to vary between cultures. Wagar and Cohen (2003) compared Euro-Canadians and Asian-Canadians using a paradigm that cued referential memory with three questions. Each of the questions corresponded to an encoding level – self-reference (“Does this word describe you?”), other-reference (“Does this word describe your best friend?”), and word structure (“Is the first letter a vowel?”). Each question was followed by the display of a word that was either a personal trait or a collective trait. The task was to decide whether the trait words applied to the cued questions. Later, a memory test on what words appeared in the previous phase was administered. Results showed that Euro-Canadian participants responded fastest to words encoded in self-reference regardless of trait type. Self-referenced words were also significantly quicker in response than words encoded in other-reference and vowel structure. The Asian Canadians, however, were only faster to recognize collective traits, but much slower with personal traits, when both were encoded in self-reference. This indicated a strong context-dependent description of the self in Asian Canadians.

Anecdotal memories were also different across the East and the West. Using a self-reported questionnaire, Wang (2001) asked participants to report their earliest childhood memory. American participants reported specific memories that were focused more on individual events (e.g., “when I was 4, I got stung by a bee”) with elaborate and expressive descriptions of emotions and personal experiences. On the other hand, the Chinese memories were more about routine activities within the family or neighborhood (e.g., “my mum took me to school everyday”), with descriptions that focused on significant others or the relationship with others.

Cross-culture variations were also found in self-face recognition. Sui et al. (2009) conducted a study to examine self-face processing using event-related potentials (ERPs) in British and Chinese participants. During the study, participants were asked to make judgments about the orientation of target faces that was either self-face or a familiar face. Behaviorally, the British participants showed a larger self-advantage than Chinese participants, but performance was faster and more accurate to self-faces than to familiar faces in both British and Chinese participants. ERP analyses revealed larger activity at 280–340 ms in the anterior N2 component for self-faces than familiar faces in British participants, while the Chinese showed reduced anterior N2 amplitudes to self-faces than familiar faces. This suggests that self-advantage is universal, but people from different cultural backgrounds develop different strategies to fit with the environment.

The neural representation of the self also differs across culture. Zhu et al. (2007) measured brain activity using fMRI as participants performed a trait judgment task. Similar to the memory test mentioned above, one of three questions was presented to cue the person to be judged – self, mother, and public figure (other). A null condition was presented between the judgment tasks where participants viewed rows of asterisks. Following the cue question, a trait adjective was presented and the participant decided whether the adjective described the cued person. Results illustrated that relative to the null condition, Western participants showed increased activations in the medial prefrontal cortex (MPFC) in response to self-judgments and reduced MPFC activities to mother-judgments. Unlike their Western counterparts, the Chinese showed enhanced MPFC activity in response to both self-judgments as well as mother-judgments when compared with other-judgments and null condition. These results indicated that while the MPFC is involved in self-representation in both cultures, MPFC also represented mother in Chinese participants.

In a similar study, Chiao et al. (2009) found different degrees of neural activation within the anterior rostral regions of MPFC due to cultural contrasts. Results found individualists, as revealed by the Self-Construal Scale, showed greater activation in the MPFC in response to general self-descriptions (traits that describe the participant in general) than contextual self-descriptions (traits that describe the participant only under certain conditions such as “when you are talking to your mother”), while collectivists demonstrated increased activation for contextual self-description than general self-description. These findings gave evidence that different cultural values have led to the neural unification or separation of the self in relation to close others.

Nevertheless, the self-bias effect has been consistently found in previous studies. Most results consistently showed a prioritization effect when processing self-relevant information. This bias toward self-stimuli was particularly robust in the perceptual matching task. Sui et al. (2012) designed a simple shape (i.e., triangle, square, or circle) and label (i.e., self, friend, or stranger) matching task. Participants were asked to learn to associate a shape with a label (e.g., triangle is you, square is your best friend, and circle is a stranger) and were tested by making judgments on whether the shape and label shown on screen matched the associations previously learnt. It was shown that responses were faster and more accurate to the self-associations than to friend and stranger associations.

In collaboration with the University of Hong Kong, the current study was conceived to test the cultural differences in self bias between participants from Hong Kong (HK) and the United Kingdom (UK). Though previous studies have examined the self-bias effect in cognitive processes such as memory and high-level decision-making (Cunningham et al., 2008; Turk et al., 2008), relatively few has examined low-level processes. Moreover, previous research often used stimuli that were highly familiar to the participant (e.g., one’s own face or familiar faces). The current study focused on low-level processing in perceptual matching by using geometric shapes as stimuli. Geometric shapes such as triangle, circle, and square are commonly found in both Western and Eastern cultures. This means that confounds such as familiarity (in studies using familiar faces as stimuli) can be controlled by making associations with geometric shapes. Additionally, geometric shapes also control for any cultural predispositions or self-relevant information carried by the stimuli prior to learning the associations. Thus, the perceptual matching paradigm (Sui et al., 2012) improves the validity of studies that make comparisons between variables.

The aim of the current study was to examine the difference in the relationship between self and others in independent and interdependent cultures using the perceptual matching paradigm. In interdependent cultures, the self-identity is embedded among the identity of family and friends, and the idea of an individual self is less pronounced. As a result, it was expected that one’s reaction time (RT) to self and friend stimuli should demonstrate a smaller difference because the friend-stimuli are a reflection of the self (see Figure 1B). In contrast, individualistic cultures promote an autonomous self, which should be reflected in a stronger sense of the individual, resulting in a faster response to the self when compared to other associations (see Figure 1A). Based on these ideas, it was hypothesized that the self-bias effect relative to friend would be significantly smaller in HK than in UK participants in perceptual matching.

## Materials and Methods

### Participants

Altogether 56^1^ volunteers took part in this study. Of these, 32 healthy Caucasian volunteers (10 male, 18–35 years of age, mean age ± standard deviation = 22.22 ± 4.51) were recruited in the UK and tested at the University of Oxford; 24 healthy Chinese volunteers (five male, 18 to 24 years of age, mean age ± standard deviation = 19.92 ± 1.91) were recruited in Hong Kong and tested at the University of Hong Kong. Hong Kong participants were bilingual and had not previously studied overseas for more than 1 year. All participants were right-handed and had normal or corrected-to-normal vision. Informed consent was obtained from all participants prior to the experiment. The procedure used in this experiment was ethically approved by the University of Oxford Central University Research Ethics Committee and the University of Hong Kong Human Research Ethics Committee.

### Stimuli and Materials

The computer task was displayed on different sized monitors, with different degrees of visual angle, in the UK and HK studies. In the UK, a white fixation cross was presented at the center of the screen at 0.8° × 0.8° of visual angle. Then, one of three geometric shapes (triangle, square, or circle) was presented above the fixation cross at 3.8° × 3.8° of visual angle, and one of three personal labels (you, friend, or stranger) was presented below the fixation cross at 3.1/3.6° × 1.6° of visual angle. The association of shapes with labels was counterbalanced across participants. The distance between the shape/label to the fixation cross was 3.5° of visual angle. All stimuli were presented in a gray background on a 23-in monitor (1920 × 1400 at 60 Hz). The program was run on a PC using E-prime software (version 2.0). All stimuli were consistent with those used in the Sui et al.’s (2012) study. In Hong Kong, the white fixation cross was presented at 0.4° × 0.4° of visual angle. The three geometric shapes were presented at 3.3° × 3.3° of visual angle, and the three labels were presented at 2.0/4.3° × 0.9° of visual angel. The distance between the shape and the fixation cross was 1.8°, and the distance between the label and the fixation cross was 1°. Stimuli were presented in a gray background on a 17-in monitor (1024 × 768 at 60 Hz). The program was run on a PC using E-prime software (version 2.0).

To determine the effects of testing using different parameters, 12 participants in the UK were tested using the HK specifications. However, I was unable to locate a 17-in monitor, so a 13-in monitor was used instead to determine whether the smaller screen affected the results. All stimuli presented were identical to the ones used in HK. A mixed design ANOVA was performed within the UK participants using the group with different parameters as a between-subject variable. No significant differences were found between the two groups, eliminating the parameters as a confounding variable. As a result, data from all participants were included in the final analyses.

Each participant completed a word-search task at the beginning of the experiment, which consisted of two short texts with no pronouns that described trips to tourist destinations. The reason behind this was that this experiment was part of a larger project. The goal of the project was to examine the effects of culture on the modulation of self-bias. The current experiment focused on the default cultural framework and its effects on the self-bias effect, while a second part of the project investigated the effects of cultural priming on the modulation of the self-bias effect. Previous studies have shown that pronouns such as “I” or “we” can successfully prime independent and interdependent self-construals (e.g., Brewer and Gardner, 1996; Sui and Han, 2007). The other part of project with priming manipulations used texts that contain pronouns such as “I” or “we.” For procedural consistency within the project, the word-search task in this particular experiment excluded the use of pronouns to make sure that the participants were tested at baseline where no cultural priming would occur as a result of pronoun words such as “I” or “we.”

Self-reported questionnaires were implemented at the end of the experiment to measure the relationship between behavioral responses and trait characteristics. This included the Self-Construal Scale (Singelis, 1994) and the Individualism and Collectivism Scale (Singelis et al., 1995).

### Procedure

**FIGURE 2:**
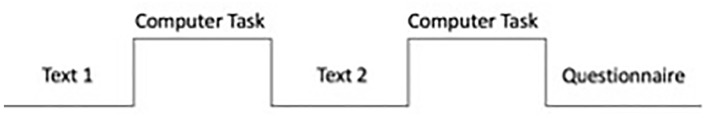
Task procedure alternating between word-search task and computer task.

Each participant conducted a word-search task and a computer-based matching task twice (see Figure 2). A questionnaire booklet was completed at the end of the study. The purpose of the word-search task was to ensure that each participant performed the computer task in their default cultural frame of mind – that is independence for UK participants and interdependence for HK participants. The word-search task consisted of two short texts with no pronouns that described a tourist destination (Sui and Han, 2007). This made sure that the participants were tested at baseline where no cultural priming would occur as a result of pronouns such as “I” or “we.” Participants were instructed to read each text and circle target nouns in the text (such as *park, area, pyramid, giza, sphinx*). The number of target words was the same for both texts. Verbal instructions were given for participants to read each text three times to make sure all target words were found. After each text, participants subsequently performed a perceptual matching task on the computer.

**FIGURE 3:**
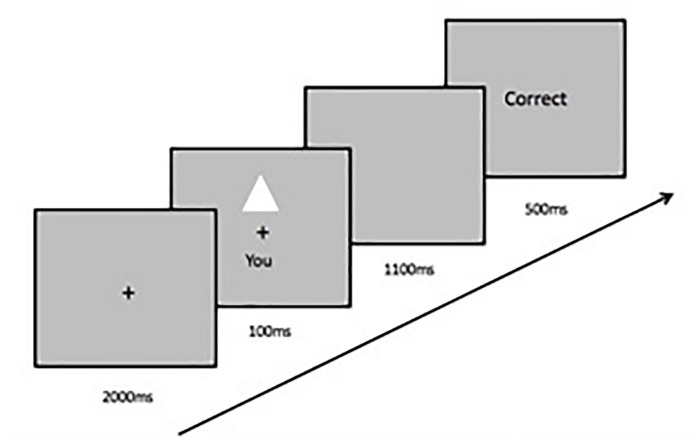
The trial procedure and an example of the stimuli for the computer task.

For the computer task, participants first learned to associate a shape with a label. For example, the triangle represents you, the square represents your best friend, and the circle represents a stranger. Verbal and oral instructions were given as the participants learned the correct associations, though shape images were not presented at this stage. The learned associations remained the same throughout the two computer tasks for each participant. At the beginning of each trial, a fixation cross was presented at the center of the screen for 2000 ms, followed by a pair of a shape with a label above and below the fixation cross for 100 ms. The shape and label either matched the associations previously learned or was a recombination of a shape with a label randomly generated by the computer. Next, the screen remained blank for 1100 ms, during which time participants had to judge whether the shape-label pair matched or not by pressing one of the two buttons as quickly and accurately as possible with one of two index fingers (see Figure 3). Following each response, feedback (green “Correct” or red “Incorrect”) was given on the screen for 500 ms at the end of each trial. If no response was given within the 1100 ms window, the feedback “Too Slow!” was displayed in yellow to prompt faster responses. Feedback on overall accuracy was provided at the end of each block. Participants performed nine practice trials and three blocks of 60 trials following each word-search task. This cycle of word-search task and computer task was repeated twice. After completing all the word-search tasks and the computer-based tasks, participants filled out the questionnaire booklet (see Figure 2).

### Experimental Design and Data Analyses

Reaction times (based on the correct responses) from the UK and HK data were normalized by calculating the difference between two associations divided by the sum of the same two associations [i.e., (A−B)/(A+B)] to equate for possible group differences in the range of RTs. This method of normalizing the results makes comparisons between two associations, which is in line with the goal of this experiment. Results were reported in normalized RTs for both match trials and mismatch trials. The results of the ANOVAs on the normalized RTs between self and friend and the normalized RTs between self and stranger were reported. Finally, analyses of d-prime were also performed. Holm–Bonferroni corrections were applied to all multiple comparisons (Holm, 1979). See Supplementary Table S1 for raw data.

## Results

### RTs on Match Trials

Since this study mainly focused on examining the difference between self and others, mixed-design ANOVAs were performed on the normalized RTs between self and friend and between self and stranger. The type of normalized RTs (between self and friend or between self and stranger) was used as the within-subjects variable and culture group (HK vs. UK) as the between-subjects variable. A significant main effect of normalized RTs type was found, *F*(1,54) = 6.28, *p* < 0.05, η^2^ = 0.10 (see Table 2). Pairwise comparison showed a larger difference in the normalized RTs between self and stranger than between self and friend (*p* < 0.05) regardless of culture group. No significant main effect of culture group was found, *F*(1,54) = 2.15, *p* = 0.15. A significant interaction between the type of normalized RTs and culture group was found, *F*(1,54) = 4.48, *p* < 0.05, η^2^ = 0.08 (see Table 1 for mean match RTs and Table 2 for mean normalized match RTs).

**TABLE 1:** Mean RTs (ms) and standard deviations (in brackets) for match trials as a function of association and culture group.

**Associations**	**UK**	**HK**
Self	632 (68)	649 (59)
Friend	695 (64)	706 (52)
Stranger	722 (56)	709 (62)
		

**TABLE 2:** Mean normalized RTs (ms) and standard deviations (in brackets) for match trials as a function of bias and culture group.

**Normalized RTs**	**UK**	**HK**	**Mean**
Self and friend	0.05 (0.04)	0.04 (0.03)	0.05 (0.04)
Self and stranger	0.07 (0.04)	0.04 (0.04)	0.06 (0.04)

**FIGURE 4:**
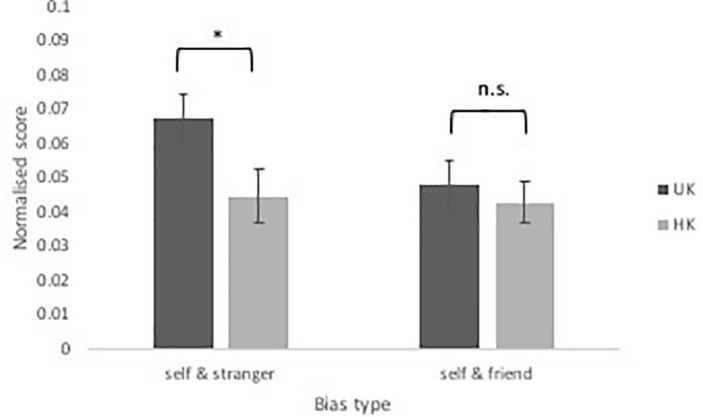
Interaction between normalized RTs and culture group. Error bars represent one standard error. Significant differences are marked with “^*^.”

Independent samples *t*-tests were performed to decompose the two-way interaction between the type of normalized RTs and culture group. A significant difference in the normalized RTs between self and stranger was found between the UK and the HK participants, *t*(54) = 2.12, *p* = 0.039, *dz =* 0.75 (see Table 2 and Figure 4). The normalized RTs between self and friend, however, was not significantly different between the UK and HK participants, *t*(54) = 0.50, *p* = 0.617, *dz =* 0.28 (see Table 2 and Figure 4). Though different from the initial hypothesis, a difference in self-bias (relative to stranger) was still identified between the two culture groups.

Additionally, one sample *t*-tests were performed on the normalized RTs to examine whether a significant self-bias effect was evident as previously demonstrated by Sui et al. (2012). Normalized RTs from both culture groups were compared against 0, which revealed significant effects of self-bias in both normalized scores between self and friend, UK: *t*(31) = 6.49, *p* < 0.001, *d =* 1.15, HK: *t*(23) = 7.19, *p* < 0.001, *d =* 1.47, and between self and stranger, UK: *t*(31) = 9.24, *p* < 0.001, *d =* 1.63, HK: *t*(23) = 5.69, *p* < 0.001, *d =* 1.16. This means that a culturally independent self-bias effect was observed in both UK and HK participants.

### RTs on Mismatch Trials

Data from shape-based mismatch trials were also analyzed. A mixed-design ANOVA was carried out with one within-subjects variable – type of normalized RTs (between self and friend or between self and stranger) – and one between-subjects variable – culture group (HK or UK). This revealed no significant main effect of normalized RT type, *F*(1,54) = 0.72, *p* = 0.40, nor culture group, *F*(1,54) = 1.81, *p* = 0.18. No significant interaction was found between the type of normalized RTs and culture group, *F*(1,54) = 0.37, *p* = 0.55 (see Table 3 for mean mismatch RTs and Table 4 for mean normalized mismatch RTs). This suggested that the responses were not significantly different between the shape-based mismatch associations across the two culture groups.

**TABLE 3:** Mean RTs (ms) and standard deviations (in brackets) for mismatch trials as a function of association and culture group.

**Associations**	**UK**	**HK**
Self	754 (63)	747 (57)
Friend	754 (61)	737 (67)
Stranger	746 (58)	735 (57)

**TABLE 4:** Mean normalized RTs and standard deviations (in brackets) for mismatch trials as a function of bias and culture group.

**Biases**	**UK**	**HK**	**Mean**
Self and friend	0.00 (0.02)	−0.01 (0.02)	−0.00 (0.02)
Self and stranger	−0.01 (0.02)	−0.01 (0.01)	−0.01 (0.02)

### D-Prime

Using the Green and Swets (1966) formula, d-prime was calculated for each participant to determine their sensitivity to correct and incorrect associations across both match and mismatch shape-label associations. This sensitivity refers to how difficult it is for the participant to discriminate the target stimuli (i.e., match trials) from background noise (i.e., mismatch trials) and is calculated using the accuracy from the match and mismatch conditions.

A mixed-design ANOVA was performed on the d-prime values, using the shape-label association (self, friend, or stranger) as the within-subjects variable and culture group (HK or UK) as the between-subjects factor (see Table 5). The analysis revealed a significant main effect of shape-label association, *F*(2,108) = 16.71, *p* < 0.001, η^2^ = 0.24. Pairwise comparison of the shape-label associations indicated that d-prime for the self-association was significant higher than for friend (*p* < 0.001), and stranger associations (*p* < 0.001). Friend and stranger associations were not significantly different from each other (*p =* 1.00). There was also a significant main effect of culture group, *F*(1,54) = 4.28, *p* < 0.05, η^2^ = 0.07. Larger d-prime was found in the UK participants than HK participants (*p* = 0.05) (see Table 5). The interaction between the shape-label association and culture group was not significant, *F*(2,108) = 0.51, *p* = 0.60.

**TABLE 5:** Mean d-prime and standard deviations (in brackets) for both match trials and mismatch trials as a function of association and culture group.

**Associations**	**UK**	**HK**	**Mean**
Self	2.76 (0.83)	2.12 (0.98)	2.48 (0.94)
Friend	2.19 (0.90)	1.77 (0.90)	2.01 (0.91)
Stranger	2.15 (1.10)	1.56 (1.02)	1.90 (1.10)
Mean	2.37 (0.10)	1.82 (0.06)	

### Questionnaires Analyses

Correlation analyses were conducted to examine whether the self-bias effect (in normalized RT scores and d-prime) correlated with questionnaire measures for independent and interdependent self-construal. Data for the following questionnaires were collected – Individualism and Collectivism Scale (Singelis et al., 1995), Self-Construal Scale (Singelis, 1994). However, no significant results were found between the behavioral responses from the computer task and measures of independent and interdependent self from either questionnaires (please see Table 6 for responses from the questionnaires). The lack of findings will be discussed in the following section.

**TABLE 6:** Mean scores and standard deviations (in brackets) for the Individualism and Collectivism Scale and the Self-Construal Scale reported by UK and HK participants.

**Questionnaire measure**	**UK**	**HK**
Individualism	112.72 (6.76)	67.38 (9.43)
Collectivism	127.41 (7.75)	76.38 (8.09)
Independence	71.63 (10.27)	68.71 (8.13)
Interdependence	70.41 (12.46)	73.29 (8.40)

## Discussion

This study explored the cultural influence on the self-bias effect in HK and UK participants through the perceptual matching paradigm. The aim of this study was to identify the differences in cultural background as a modulating factor for the self-bias effect. The initial hypothesis was that due to cultural differences in the emphasis on independent and interdependent self, the self-bias effect relative to friend would be larger in the UK participants than HK participants. Though the results in RTs were slightly different from this hypothesis, a cultural variation was still identified between the two cultural groups: the UK participants demonstrated a larger self-bias effect relative to strangers than the HK participants. This difference in the self-bias effect relative to strangers was an indication that independent and interdependent cultural frameworks can modulate the magnitude of the self-bias effect in RTs. In contrast, d-prime results showed no cultural modulation on the self-bias effect. The d-prime results from both culture groups demonstrated the robustness of the self-bias effect – there was a significant advantage for the self-association than for friend and stranger associations in both HK and UK participants. This confirmed the prioritization in the processing of self-relevant information, consistent with previous research (e.g., Frings and Wentura, 2014; Mattan et al., 2015). However, no significant cultural differences in d-prime were observed between the two groups.

However, the UK participants were much more sensitive to all of the stimuli than HK participants, resulting in higher overall d-prime in the UK than in HK participants. The lower d-prime score may be due to the fact that the HK participants found the task more difficult. Previous research found that interdependent samples process objects with reference to the context or background more than Western samples (e.g., Masuda and Nisbett, 2001, 2006; Wang and Ross, 2005). It is possible that this strategy is not as well suited for the interdependent subjects of this particular task where the stimuli were presented on a blank background without context. In contrast, Western samples have an advantage over the Eastern samples as they process target objects individually.

Alternatively, the lower d-prime performance in the HK participants may have been a result of testing in a second language (English). One of the criteria for the HK participants was that they must not have studied more than 1 year abroad. Although this controls for the level of exposure to independent cultures, it also suggests that the participants may not be fluent in the English language. In which case, individuals may have more difficulty detecting the English stimuli. Future research should be conducted to test another group of non-native English speakers using English stimuli and use language as a between-subjects factor to see if there are any significant integration between English as a second language and task performance. Despite these limitations, the results of our experiment identified a difference in the self-bias effect between the two cultural groups.

Although no significant results were found between the questionnaire measures and the self-bias effect, it is important to keep in mind that the self is an ambiguous and abstract concept that is difficult to measure (Grace and Cramer, 2003). Many have questioned the reliability of questionnaire measures due to inconsistent results. For example, Harb and Smith (2008) criticize self-construal scales for not providing references to specific contexts in the interdependent measures when interdependent individuals are particularly sensitive to contextual cues. The environment is crucial to providing messages that elicit access to independent or interdependent self-construals (Sorensen and Oyserman, 2013). Large-scale studies across 30 nations also suggested that the Self-Construal Scale is unstable in structure within and across cultures (Levine et al., 2003; Georgas et al., 2006). Moreover, the number of questionnaire responses in this study may be too limited to produce enough statistical power to the correlation analyses. It is also important to note that western and eastern samples are not restricted to being only independent or interdependent. For example, although Americans were typically attributed to being more individualistic than people from other cultures, they are not less collectivistic than East Asians (Takano and Osaka, 1999; Oyserman et al., 2002). Independent and interdependent cultures are, in fact, two parallel dimensions that are not negatively correlated (Ng and Lai, 2011), which means that a higher measure of independence does not indicate a lower measure of interdependence, and vice versa. The results from the questionnaires in this study also confirmed this in that responses from both UK and HK participants showed similar values on independent and interdependent scores in the Self-Construal Scale and individualism and collectivism scores in the Individualism and Collectivism Scale. The self is a dynamic concept that can shift and evolve based on relations and contexts (Nisbett et al., 2001), and thus makes self-construal difficult to measure with fixed scales.

The findings of this experiment indicated that cultural variations play a role in the way humans process information about ourselves and unfamiliar others. Although previous studies have addressed cross cultural comparisons, the significance of this experiment lies in utilizing the shape-label paradigm, which included simple geometric shapes as stimuli (Sui et al., 2012). Previously, experiments have used stimuli such as faces (e.g., Keenan et al., 1999; Sui et al., 2009) and texts (e.g., Zhang and Mittal, 2007; Chiao et al., 2009) to study self-construal styles. These stimuli may introduce confounding variables because visual familiarity of faces, as well as complex language processes, are difficult to control. Geometric shapes, however, are universal in both Western and Eastern cultures, which makes it less susceptible to any cultural predispositions. The association of familiar and unfamiliar persons with geometric shapes eliminates the confounding effect of visual familiarity with faces, and removes translational and language problems produced in complex texts. The use of geometric shapes may also eliminate any self-relevant information carried by the stimuli prior to learning the association. Thus, this paradigm potentially improves the validity of the cross-cultural comparisons and provides more comparable data without the introduction of prior knowledge.

Some limitations of this experiment are as follows. First, HK participants are considered more bicultural than other East Asian cultures. Hong Kong has been the confluence of both Chinese and Western cultures for nearly two centuries. The Hong Kong students recruited in this study may be more representative of a bicultural sub-group than the typical East Asian interdependent group. This may explain the lack of a decrease in self-bias relative to friend as was initially hypothesized, though further research is needed to fully explain this phenomenon.

Second, the experiment was administered in English in both the UK and the HK groups. Usually, experiments that use languages as stimuli are administered in the native tongue to avoid the effects of cultural priming. Participants who used an independent language (i.e., English) show decreased cognitive accessibility of the interdependent self compared to those who used a interdependent language (i.e., Chinese) (Trafimow et al., 1997; Wang et al., 2010). Ross et al. (2002) also reported that bicultural participants were more likely to report more favorable self-statements when writing in English than in Chinese. According to Kemmelmeier and Cheng (2004), language priming effects primarily occurred for self-construals that were not already salient in the respondents’ culture. Despite this, the results of this experiment are still quite robust. Therefore, it is unclear to what extent the language cue has affected these results. Notably, the results showed the cultural effect even with these limitations.

Third, it is impossible to determine how much of the observed difference in this study could be attributed to cultural differences. For example, the reaction to strangers in HK participants could potentially be explained by the high-density population in Hong Kong. People may simply be more aware of others due to living in crowded spaces, as opposed to the British living in relative spacious environments. Other potential explanations could be living habits, genetics, etc. However, whether the significant findings were due to the study manipulations is a problem that is commonly faced by all researchers. Thus, it is important to keep in mind that we should be careful when drawing conclusions from study results. In the case of this study, interdependent culture should be considered a potential explanation for the results and not a definitive cause, especially when the questionnaire data do not support it.

This experiment provides some interesting future research questions. First, it would be interesting to explore the extent of the self-bias effect in relation to family members in cultures which possess different family values. Moreover, the strength of the self-bias against other members such as siblings, spouse, or children have yet to be examined. Interdependent cultures would suggest a stronger embeddedness of the self in one’s family members than independent cultures. It would be interesting to examine whether the closeness of one’s family member predicts the strength of the self-bias effect and how this effect changes due to cultural values.

Second, it is important to keep in mind that individual differences can exist within culture groups. Some people from independent cultures may be more interdependent and some from interdependent cultures may be more independent. The concept of the self, whether independent or interdependent, is a dynamic concept that can change depending on many factors such as social context (Markus and Kunda, 1986; Markus and Wurf, 1987; Oyserman et al., 2009; Colzato et al., 2012). Hence, future studies should take into consideration that cultural background is not the determining factor on the magnitude of the self-bias effect.

## Conclusion

The present study investigated the impact of culture on self and other processing through a new paradigm. The results revealed that compared to their UK counterparts, HK participants showed reduced self-bias in relation to strangers. This supports the concept that interdependent self-construal style recognizes the self as an entity in relation to others while the individualist self is a single independent entity. Hence, cultural background can modulate the self-bias effect.

## Ethics Statement

This study was carried out in accordance with the recommendations of the University of Oxford Central University Research Ethics Committee and the University of Hong Kong Human Research Ethics Committee with written informed consent from all subjects. All subjects gave written informed consent in accordance with the Declaration of Helsinki. The protocol was approved by the University of Oxford Central University Research Ethics Committee and the University of Hong Kong Human Research Ethics Committee.

## Author Contributions

MJ, YS, JH, JS, and GH contributed conception and design of the study. MJ organized the database. MJ, SW, and HC performed the statistical analysis. MJ wrote the manuscript. All authors contributed to manuscript revision, read and approved the submitted version.

## Conflict of Interest Statement

The authors declare that the research was conducted in the absence of any commercial or financial relationships that could be construed as a potential conflict of interest.

## Footnotes

1 Based on the effect size from previous research (e.g., Sui et al., 2012), a minimum of 26 subjects were required to achieve *power* = 0.80, *a* = 0.05.

## Supplementary Material

TABLE S1 Raw data in normalized RTs and d-prime.
